# Neoliberalism is not dead – On political implications of Covid-19

**DOI:** 10.1177/0309816820982381

**Published:** 2021-06

**Authors:** Miloš Šumonja

**Affiliations:** Faculty of Education, University of Novi Sad, Serbia

**Keywords:** authoritarianism, coronavirus crisis, Covid-19, far-right, neoliberalism, state

## Abstract

The news is old – neoliberalism is dead for good, but this time, even *Financial Times* knows it. Obituaries claim that it had died from the coronavirus, as the state, not the markets, have had to save both the people and the economy. The argument of the article is that these academic and media interpretations of ‘emergency Keynesianism’ misidentify neoliberalism with its anti-statist rhetoric. For neoliberalism is, and has always been, about ‘the free market and the strong state’. In fact, rather than waning in the face of the coronavirus crisis, neoliberal states around the world are using the ongoing ‘war against the virus’ to strengthen their right-hand grip on the conditions of the working classes.

## Introduction

The coronavirus crisis has dominated the world news for the last few months and is likely to do so for the foreseeable future. Public and mental health, the value and meaning of work, science, distribution of resources, democracy and civil rights, human’s place in nature, the character of education – the crisis seems to have influenced the relations of capital’s social reproduction at each structural level. While the chain of consequences continues to unravel with the relentless speed of the daily news circle, the events themselves draw the patterns of inequality behind the growing human suffering for which no sensible explanation can be given within the confines of free market ideology. That, one might argue, is how the wheels of history start to turn, with old certainties disappearing overnight, along with three meals on the kitchen table, face masks and hospital beds.

The media reports on the actions undertaken by authorities which break every rule in the neoclassical playbook. Worldwide, states are spending massively to protect society from a combination of a public health and economic disaster. At the moment, almost nobody is asking, ‘How are we going to pay for it?’ Moreover, a prominent right-wing commentator for the conservative *Daily Telegraph* urged British PM Boris Johnson ‘to embrace socialism immediately’ in order to avert it (Evans-Pritchard 2020). It is not just that Covid-19 has led Johnson to wittingly contradict his Tory predecessor Margaret Thatcher and recognise ‘there really is such a thing as society’ (*The Guardian* 2020). It appears that the ‘magic money tree’ also exists.

Commenting on the fact that global central banks had by mid-April, poured 8 trillion dollars to stave off the economic collapse, George Saravelos, Deutsche Bank’s global head of foreign exchange research, hardly a radical thinker, concluded that ‘there is no such thing as a free market anymore’ (Saravelos 2020). Along similar lines, the business-friendly *Economist* predicts that ‘the state is likely to play a very different role in the economy’, noting that ‘history suggests that the effects will be permanent’ (*The Economist* 2020). Adding his voice to the heresy, Emmanuel Macron, a former banker turned French President, declared in an interview to *The Financial Times* that it is ‘time to think the unthinkable . . . while socialism in one country may not have worked, the unthinkable in all countries forces us to reinvent ourselves’ (Macron 2020). The newspaper of the City of London predicts that ‘[r]adical reforms – reversing the prevailing policy direction of the last four decades – will need to be put on the table’ (*The Financial Times* 2020). Small wonder then that the ideological mood of the public outside the church of neoliberalism appears even worse to a true believer such as a writer for the billionaires’ magazine *Forbes*, who warns:

[N]ow is the time to be extra vigilant: 12 years ago, anti-capitalists succeeded in reframing the financial crisis – wrongly – as a crisis of capitalism. The false narrative that the financial crisis is a result of market failure and deregulation has since become firmly established in the minds of the population at large. And now left-wing intellectuals are again doing their utmost to reframe the corona crisis to justify their calls for the all-powerful state. Unfortunately, the chances that they could succeed are very high indeed. (Zitelmann 2020)

The consensus is indeed growing across the political spectrum that ‘this time truly is different’ (Reinhart 2020): that ‘the state is back’ with a vengeance against markets (Golub 2020; Hameiri 2020; Rodik 2020): that we are witnessing ‘the death of neoliberalism’ (Avineri 2020; Cherkaoui 2020; Lent 2020; Saad-Filho 2020; Wong 2020). With 2008 in mind, one might wonder – dead again?

What this article will argue is that although it is true that the coronavirus crisis has thus far presented hard evidence that confirm the Marxist hypothesis about suicidal tendencies of untamed capitalism, it would be a mistake to conclude that the neoliberal era is over just on account of the ‘emergency Keynesianism’ that governments around the world are resorting to. The point is not to deny that policies like the albeit temporary and selective nationalisation of wages in the United Kingdom (up to 80%; Partington 2020) or requisitioning of private hospitals in Spain imply the public recognition that the very survival of society depends on there being alternatives to markets (Jäger & Klein 2020). However, these actions mean that we ‘can watch neoliberalism collapsing in real time‘ (Monbiot 2020) only if understood in terms of the state versus markets dichotomy, as a necessarily progressive shift in power between them in favour of the former. To explain the process that includes ‘the morbid symptoms’, like the suspension of parliamentary democracy in Hungary or courts in Israel (Gebrekidan 2020), as much as any ostensibly left-wing policy, we should think of it in terms of capital’s struggle to govern the working class. This includes conditions of emergency, when the state authorises itself to do ‘whatever it takes’ to save the existing social order from breaking down. In other words, we should remember that the strong hand of state, whose supposed return in action is cheered and feared today, has actually been the organising force of neoliberal assault on all political obstacles to the profitability of capital accumulation.

### The neoliberal pandemic – the causes of crisis

From the viewpoint suggested above, the results of capital’s totalising effort to commodify and eat away its own social and natural preconditions appear as the structural causes of the pandemic, which thus represents a potentially fatal internal crisis of neoliberalism. The crisis is not, as one might conclude from the establishment’s ‘going back to normal’ narrative, an exogenous shock to an otherwise functional system. A Black Swan-type event (Smith 2020), or a meteorite of history which can only be ascribed to a foreign entity is how capitalism’s ideological defence in the West already projects its own contradictions: to the contingency of nature (Joye 2020); then to virus-producing China (on 1 February, the German newsmagazine *Der Spiegel* run a cover page with a headline ‘Coronavirus – Made in China’; *Der Spiegel* 2020); to a virus-carrying immigrant; and in a final act of inversion of reality, to the class enemy within (as in the case of Amazon warehouse employee, Chris Smalls, who organised his coworkers to demand safer working conditions amid the pandemic, only to find himself fired because of – breaching of the safety measures; see Bellafante 2020).

The interventions from the Left highlight at least three constitutive connections between neoliberalism and the pandemic. According to Rob Wallace (2020a, 2020b) evolutionary biologist and phylogeographer, the increased appearance of corona viruses like SARS, MERS or Covid-19 in the human population is a predictable outcome of agroindustry’s devastating impact on natural ecosystems rather than a series of isolated incidents. What is seen is an interplay between industrial production of food and a growing market for exotic wild food. The multinationals’ land-grab and deforestation push the increasing capitalised wildlife deeper into the remaining primary ecosystems. This enables the spillover of previously boxed-in pathogens to human communities that are forced to breach the natural barrier between them while working:

Capital is spearheading land grabs into the last of primary forest and smallholder-held farmland worldwide . . . As industrial production – hog, poultry, and like – expand into primary forest, it places pressure on wild food operators to dredge further into the forest for source populations, increasing the interface with, and spillover of, new pathogens, including Covid-19. (Wallace 2020a).

As for the speed at which the virus has spread, the unprecedented physical connectivity in the word of global supply chains and low-cost flying was not the only contributing social factor. It should not be forgotten that the initial reaction from most governments to the outbreak was an exercise in ‘epidemiological neoliberalism’ (Frey 2020). This policy bluntly exposed the politics of the whole project: pretend to do nothing while making sure that the ‘natural laws’ of markets keep functioning, even if it means allowing people to get sick and die from ‘just another flu’. Encapsulated in social-Darwinian ‘survival of the fittest’ notion of ‘herd immunity’, this solution in practice consisted of voluntary behavioural guidelines – business as usual, just wash your hands and keep your distance. This, in effect, turned a social problem into an individual matter, thus shaking off any responsibility the authorities had for the public health crisis.

However, with the numbers of infected inevitably rising in consequence, the genocidal charade backfired, not just because the public lost faith in neoliberal crisis management but also because the markets lost confidence in it. In the United States, for example, President Donald Trump changed his approach only during the March stock crash. Then, in the wake of what was becoming the greatest pandemic since the Spanish Flu, the disastrous effects of 40 years of neoliberal privatisation of public health institutions were revealed. That is, the lack of staff and material capacities in underfunded state hospitals, and the complete inability of the private for-profit health industry to provide even the most basic medical equipment and treatment in the time of social need. To illustrate the point: in Italy, one of the countries hardest hit by the pandemic, austerity cuts in the national health system resulted in an extraordinary 50% reduction in hospital beds between 1997 and 2015, and 46,000 less hospital employees from 2009 to 2017 (Autore & Corizzo 2020). Add the outsourcing of medical production in search for cheap labour and that health companies have no commercial interest in preparing for or preventing emergency situations – in keeping hospital beds empty and magazines stocked with face masks and gloves, or investing in vaccine development – and you get a centennial public health crisis.

In response to the crisis, the resources of national states have been mobilised in full force, mandatory lockdowns imposed, and branches of industry told what to produce. However, with the majority of workers not working, global supply chains broke, demand collapsed, production fell, company revenues sharply dropped and stock markets plunged. The IMF projects global growth to fall to -3%, a downgrade of over 6 percentage points from January 2020, which makes the ongoing capitalist crisis ‘the worst economic downturn since the Great Depression’ (Gopinath 2020). Indications of what awaits the working classes are clear: in the United States, the world epicentre of both neoliberalism and the pandemic, unemployment rose to 14.7% in April, again the worst rate since the Great Depression (BBC 2020). Moreover, in August, the real unemployment rate, which includes underemployed and marginally attached to the labour market, reached 16.8% (Hindery 2020), with a significant number of disproportionately women and minority workers facing permanent job loss (Data Speaks 2020).

A number of authors on the Left, who have warned of this crisis even before Covid-19 detonated it, argued that the pandemic has brought to light the underlying weaknesses of the neoliberal capital accumulation model in terms of investment, productivity and growth. These had all been left unresolved in the aftermath of the 2008 Recession (Harvey 2020; Lapavitsas 2020; Roberts 2020). While delivering harsh austerity to people across the globe, the last decade has seen the transformation of failed ‘privatised Keynesianism’ policy regime (Crouch 2009), reliant on private debt, to a policy of a ‘quantitative easing ad infinitum’. Simply put, central banks provided cheap money to commercial banks for loans to big corporations, which used them, not to create jobs, but to buy back their own stock and so enrich their shareholders. The gravity of an increasingly exhausted real economy was bound to kick in. The gap between promised value of financial capital gains and the insufficient production of surplus value has become unsustainable even in the eyes of the investor class (Toussaint 2020). In fact, during the last quarter of 2019, the US Federal Reserve had to calm the financial markets with massive injections of liquidity. German industrial production fell to its lowest levels since 2008, as did China’s economic growth.

So, states intervened and started acting as buyer, employer and creditor of last resort. No neoliberal taboo was left unbroken: from a 1,200 dollars per person government giveaway in the United States; nationalisation of payrolls across the Europe; different credit guarantee schemes suspension of mortgage payments and additional funding (48.5 billion pounds) for the NHS, public services and charities in the United Kingdom; expanded childcare benefits for low-income parents and basic income support for the self-employed in Germany; tax and utility bill holidays, and nationalisations of ailing companies in France (International Monetary Fund [IMF] 2020); to government takeovers of Alitalia airline in Italy (Roberts & Leali 2020) and hospitals in Ireland (TheJournal.ie 2020). In a passage summing up ‘the return of the state’ interpretation of these developments, one author writes:

The age of neoliberalism, in terms of the primacy of market interests over all other social interests, is coming to an end . . . After four decades of neoliberal scepticism about the state, a long-forgotten fact is coming to the light: that nation states still have enormous creative power, if only they are willing to use it. (Saxer 2020)

### Nationalist states and neoliberal solutions

Surprisingly, after all the historical experiences from the 1970s onwards, and all the theoretical work done to explain the neoliberal counter-revolution, its common sense ‘small state’ ideology still haunts the Left, in particular Keynesian critics of austerity. Even works accentuating the conflict between capitalism and democracy, like Wolfgang Streeck’s (2014) widely read *Buying Time*, describe neoliberalism as ‘a general rollback of the state and its intervention in the markets’ (p. 124). Thus, it is worth recalling the lessons of the Marxist *State Debate* (Clarke 1991), particularly the insight that the apparent self-isolation of the state from the economy, which seems to be taken for granted in obituaries written for neoliberalism since 2008, is a function of its class nature. The modern state is capital’s home court in its struggle against the masses because it sets the limits to democracy around capitalist social relation of production – although in a way that also depends on the outcome of political and class conflict. Its welfarist incarnation projected and restricted an unprecedented egalitarian change in those relations across the post-war West to the level of distribution. Democratisation of capitalism through the Keynesian state – meaning strong trade unions, politically guaranteed full employment, redistribution of wealth and life chances to the dispossessed, and extensive social services and benefits for all citizens – was a mode of class rule based on compromise rather than outright conflict with the workers. This accumulation regime began to crumble during the late 1960s and early 1970s due to the fall of the rate of profit, when struggles over distribution started to erode the relations of production (Davidson 2018; Streeck 2014), and capital owners lost confidence in the social and democratic state of capitalism. In these circumstances, neoliberalism appeared as capital’s solution to the economic crisis of 1973, and as a long-term project of class rule.

Theoretically and practically, neoliberalism is committed not only to the protection of markets from society and democracy but also to the transformation of society in the image of markets at the expense of egalitarian democracy (Munck 2005). That explains why neoliberalism requires a strong and active state, which is capable of reorganising social relations, in particular those characteristic to its Keynesian predecessor, in a way that deepens and entrenches the inequalities of capitalism – through the crushing of the trade unions, cuts in social provision, privatisation of public industries and services, deregulation of financial markets, monetary policies predicated on price stability and so on. Therefore, the so-called ‘roll-back of the state’ is just an ideological misnomer for the process of its neoliberal restructuring in the course of the capital’s efforts to restore the rate of profit:

Projects of neoliberalisation . . . have never been synonymous with a simple diminution, or withdrawal, of the state, but instead have been variously concerned with its capture and reuse, albeit in the context of a generalized assault on social-welfarist or left-arm functions, coupled with an expansion of right-arm roles and capacities in areas like policing and surveillance. (Peck & Theodore 2019: 249)

Here, the point is to note that the coronavirus crisis is being managed by the neoliberal state, whose purpose was, and still is, ‘the creation and maintenance of a politico-economic order which actively defends itself against impulses towards greater equality and democratisation’ (Bruff 2016: 110). No doubt, the fact that it has been forced to shut down the economy and break open the Keynesian toolbox it was meant to bury forever, speaks of an organic crisis in the whole social order. For, paradoxically, in conditions of emergency, when states demonstrate their full commitment to the survival of markets, even if it means temporarily prioritising the survival of the people, the laws of capitalism show themselves as a matter of political choice, and not of natural necessity. By the same token, however, there is nothing contradictory in neoliberal ‘disaster socialism’. Depending on the future, the coronavirus crisis might, in retrospect, appear as just another capitalist crisis, an integral part of its life cycle, or a round of ‘creative destruction’ – and, therefore, another reorganisation, that is further de-democratisation of social relations. As a matter of fact, the crucial question of who is paying for the crisis is already being answered in practice.

First, contrary to the media myth that the pandemic is a ‘great equaliser’, it is actually aggravating the pre-existing inequalities. The sections of the low-paid precariat, who were not fired but deemed essential for maintaining the society during the lockdown – for example, workers in hospitals, grocery stores, warehouses, package and postal workers – have been confronted with a choice between keep working and risk getting sick, or quit and starve. These sections of the working class are mostly racialised, gendered and ethnicised around the world, which helps explain why, for example, 70% of all Covid-19 deaths in Louisiana have been African American residents. This is twice their percentage in the population of that state. Similarly, in New York, ‘19 of the 20 neighbourhoods with the lowest percentage of positive tests have been in wealthy ZIP codes’ (Buchanan et al. 2020).

As for ‘the magic money tree’, you do not have to believe those denouncing short-lived benefits for the working classes as a cover for a re-run of 2008 corporate bailout but on steroids (Slobodian 2020) – the markets themselves have given their verdict, most notably on the 2.2 trillion dollar economic rescue legislation by the US Congress on 25 March 2020. In the 10 weeks from the passing of the bill, to the day that historic unemployment numbers were announced in the United States, the stock of Blackrock, the world’s biggest private equity fund, rose by 50%. This is because its assets have been, in effect, unconditionally insured by the Fed, the world’s biggest central bank (Eisinger 2020). However, the principle of supporting capital at the expense of workers also holds in less neoliberal countries like Sweden, whose government’s rescue package covers the full costs of all sick leave – normally paid by employers (Solty 2020).

That said, it is reasonable to suppose that, as a consequence of the pandemic, national states will increase spending on the public health, possibly restoring some of the social benefits they had previously cut. It is also likely that they will strive for sovereignty in the production of vital goods like food or medications. Nevertheless, as we have witnessed during the last decade, much of neoliberalism’s resilience rests on its anti-democratic DNA allowing different mutations in the conditions of crisis. This is dependent on them being able to ‘save the capitalism from itself’ without disturbing wealth distribution, while framing the policies in market terms. Writing about the contemporary right-wing critique of austerity, Melinda Cooper (2020) revisits the economic policies of the Third Reich to point out an unsettling affinity between the radical right authoritarianism and application of anti-deflationary measures. These surface when capitalism seems to be on its knees – in line with Friedrich Hayek’s (2013) words that the state, the force of exception underpinning ‘the basic principles of free society’, may have ‘to temporarily suspend [them] when the long-run preservation of that order is itself threatened’ (p. 458). Thus, the thesis that, by treating the pandemic as an existential threat to the nation from an invisible enemy, neoliberal state has thus far used the ‘war against the virus’, to accelerate its ongoing authoritarian turn.

In its first ‘vanguard’ phase, the Thatcher-Reagan era, neoliberalism stood for a frontal all-out attack on organised labour – the perceived cause of the crisis of profitability – through the unemployment-inducing policy of sound money, austerity, and coercion, like in the case of the famous Miners’ Strike of 1984/1985 (Davidson 2017). It was authoritarian, wrapped in social conservatism, and intent on discarding egalitarian democracy. In its second ‘progressive’ phase, from the end of the Cold War up to the 2008 Great Recession, neoliberalism became both a hegemonic and global social order – due, but not exclusively, to (1) neoliberal conversion of the centre-Left parties in the West, and their abandonment of the working class as a constituency; (2) governments’ policies of giving away economic sovereignty to international financial and trade institutions; (3) its cultural legitimisation in libertarian values of 1960s social movements, recruited to denounce all collective identities as oppressive in the name of the hyper-individualised freedom of market choice (Streeck 2014).

In its present ‘authoritarian’ phase, post-2008, neoliberalism is ‘breaking with elements of formal democracy and is infringing fundamental rights’ (Oberndorfer 2015: 187). Unlike before, it is not a counter-revolutionary solution to the capitalist crisis, but an endeavour to sustain and normalise an unending condition of the capitalist crisis – it ‘is primarily defensive, an attempt to preserve the now decaying order through ever more generalised attacks on the subaltern classes . . . as permanent aspects of the political regime’ (Davidson 2017: 3). Drawing on the work of Nicolas Poulantzas (1978), Ian Bruff (2014) further explains:

Authoritarian neoliberalism does not represent a wholesale break from pre-2007 neoliberal practices, yet it is qualitatively distinct due to the way in which neoliberalism’s authoritarian tendencies . . . have come to the fore through the shift *toward* constitutional and legal mechanisms and the move *away* from seeking consent for hegemonic projects . . . [U]nder authoritarian neoliberalism dominant social groups are less interested in neutralizing resistance and dissent via concessions . . . favoring instead the explicit exclusion and marginalization of subordinate social groups. (p.116)

So, arguably, last vestiges of the façade of democracy and social libertarianism are falling off capitalism before our eyes.

Witness how ‘naturally’ the populist Right’s re-interpretation of the cult of competition – a neoliberal justification of inequality that replaces the cosmopolitanism of individualised meritocracy with national competitiveness on a global stage – lends itself to the governments’ strategy of ‘othering’ and securitizing the virus. Hungarian PM Victor Orban, always the avant-garde of Europe’s new Right, has pushed for the emergency corona bill which will enable him to indefinitely rule an EU country by decree. At the same time, he blames the Iranian students living in Budapest for the arrival of virus. Orban explained:‘We are fighting a two-front war, one front is called migration, and the other one belongs to the coronavirus, there is a logical connection between the two, as both spread with movement’ (Orban, quoted in Rohac 2020). The task of containing the Covid-19 has therefore become a project for controlling society. In the United Kingdom, the police and immigration officials have been given the power to arrest people suspected of carrying the virus (Mudde 2020). In Israel, the government invoked the pandemic to authorise the internal security agency to access the private phones of citizens. In India, the announcement of lockdown ended the protests against PM Narendra Modi’s anti-Muslim policies (Roth 2020), while all workers have been obliged to download a contact-tracing mobile app, presumably in order to determine their level of contagion risk (*Al Jazeera* 2020).

Furthermore, this kind of private-public regime, a partnership between the IT industry and increasingly authoritarian states, consistently purports to make the nation’s human capital more competitive by protecting the national health through policing of the society. The stated purpose of the neoliberal solution to the coronavirus crisis is ‘strengthening the immune system of economy’ – which is how the German right-wing party *Alternative für Deutschland* described its call for deportation of refugees. And for defunding of the gender studies in the name of ‘the knowledge that can be used for the bare reality of the labour market’ (AFD Bundestag 2020). In fact, ‘disaster socialism’ looks more like a radical form of nationalised neoliberalism that strengthens the right, not the left, hand of the state. It seeks not to alleviate but to re-establish and sharpen the social inequality by separating the healthy/productive from the unhealthy/unproductive parts of population along ethnic, racial and cultural lines.

To conclude, as a result of the coronavirus crisis, the idea of ‘a retreat back to the sort of competing forms of nationalism’ (Worth 2019: 189) appears more realistic than before. In the face of social unrest and struggles that have already begun, the argument just made could be understood as a warning to the progressive political forces not to repeat the mistakes from 2008. As this article has tried to show, the critique of the capitalist state claiming that it relates to people in a way that individualises social problems and divides the working class sounds like a truism today – more a description than an explanation of neoliberal governance of the pandemic. Hence, we should not believe that state activism in reaction to the capitalist crisis *automatically* undermines neoliberalism, and leads to a one-way road back to the national social-democracy, or, particularly in Europe, a birth of its trans-national form.

Yet, as Philip Mirowski (2014) pointed out, this anti-statist misidentification of neoliberalism has also plagued horizontalist strategies of resistance – contrary to which, the national state is anything but obsolete, as the pandemic made it clear. It seems to me that the insight offered here would best serve the radical-Left forces if it were to be followed by the realisation that at least one of the reasons why the populist revolt against ‘progressive neoliberalism’ has been much more effective from the radical-Right is its clear-eyed, although reactionary, view of the power and vitality of the national state, as a set of institutions with a degree of political plasticity. Too often to ignore, the blanket anti-statism of the contemporary social movements has betrayed practical impotence and empty radicalism, concerned with self-expression and/or ‘changing the nature of political discourse and the various spheres in which it is carried on’ (Gamson 2012). Arguably, in retrospect, that self-professed cultural change has brought more excitement to academic than ordinary discussions about politics. To be clear, it is hard to imagine any progressive social transformation that does not reappropriate ‘the language of ‘freedom’, ‘rights’, ‘democracy’, and ‘justice’ from neoliberal . . . framings’ (Bruff 2014: 127). Why should the meaning of ‘state’ be an exception in this respect, particularly in the circumstances when its ‘neoliberal framing’ is open to contestation? Its left hand is weak, but the state remains a primary political space for a collective resistance to the coming wave of anti-democratic policies. Therefore, it may be useful to consider the recently revived concept of the fight ‘in and against the state’, originally developed in the second half of the 1970s in an analysis of the contradictory relation of public employees to the British state (London-Edinburgh Weekend Return Group 1980). For, in consequence of the pandemic, the vast majority of people find themselves in a similar contradictory position of having to assert their existential interests and fundamental rights in the institutions that subvert them. This is why the state should not be ceded to the far-Right in the streets, in the ballot box, or on the level of everyday discourse.
